# Understanding the Nature of Medication Errors in an ICU with a Computerized Physician Order Entry System

**DOI:** 10.1371/journal.pone.0114243

**Published:** 2014-12-19

**Authors:** Insook Cho, Hyeok Park, Youn Jeong Choi, Mi Heui Hwang, David W. Bates

**Affiliations:** 1 Department of Nursing, Inha University, Incheon, Republic of Korea; 2 Harvard Medical School, Boston, MA, United States of America; 3 Division of General Internal Medicine, Brigham and Women's Hospital, Boston, MA, United States of America; 4 Department of Nursing, Inha University Hospital, Incheon, Republic of Korea; 5 Partners Healthcare Systems, Inc., Wellesley, MA, United States of America; D'or Institute of Research and Education, Brazil

## Abstract

**Objectives:**

We investigated incidence rates to understand the nature of medication errors potentially introduced by utilizing a computerized physician order entry (CPOE) system in the three clinical phases of the medication process: prescription, administration, and documentation.

**Methods:**

Overt observations and chart reviews were employed at two surgical intensive care units of a 950-bed tertiary teaching hospital. Ten categories of high-risk drugs prescribed over a four-month period were noted and reviewed. Error definition and classifications were adapted from previous studies for use in the present research. Incidences of medication errors in the three phases of the medication process were analyzed. In addition, nurses' responses to prescription errors were also assessed.

**Results:**

Of the 534 prescriptions issued, 286 (53.6%) included at least one error. The proportion of errors was 19.0% (58) of the 306 drug administrations, of which two-thirds were verbal orders classified as errors due to incorrectly entered prescriptions. Documentation errors occurred in 205 (82.7%) of 248 correctly performed administrations. When tracking incorrectly entered prescriptions, 93% of the errors were intercepted by nurses, but two-thirds of them were recorded as prescribed rather than administered.

**Conclusion:**

The number of errors occurring at each phase of the medication process was relatively high, despite long experience with a CPOE system. The main causes of administration errors and documentation errors were prescription errors and verbal order processes. To reduce these errors, hospital-level and unit-level efforts toward a better system are needed.

## Introduction

Medication errors are particularly hazardous for critically ill patients and can increase the risk of adverse outcomes in this population [1]. The patients in an intensive care unit (ICU) will typically experience a mean of 1.7 errors per day. Nearly all patients in an ICU will be affected by a potentially life-threatening error at some point during their stay [2]. Medication errors account for 78% of the serious medical errors in an ICU [3], [4].

Many clinicians already use a computerized physician order entry (CPOE) system in their routine patient care, and the adoption of CPOE systems and electronic health records continues to increase worldwide. The use of electronic prescriptions should make the process safer and ensure that key fields include meaningful data as well as deliver clinical decision support for critically ill patients who are receiving highly complex medications. However, these benefits do not always occur, and error rates may increase with order complexity. For example, results from a controlled laboratory study measuring the rate of prescription errors associated with inpatient CPOE found that the mean error rates ranged from 1.5% to 2.6% depending on order complexity [5]. Another study examining the effect of CPOE systems in a pediatric ICU found several types of new errors, such as incorrect infusion rates for continuous infusions and incorrect selections of multiple dosage options available for some drugs [6]. In addition, results from a retrospective study showed that new types of errors were experienced with an electronic prescription system, differing from those commonly found when using a paper system [7]. Together, these studies illustrate that any systemic change can have unintended consequences. However, little is known about how prescription errors affect the subsequent processes, how many errors reach patients, and how prescription errors are treated by nurses.

To determine the nature of medication errors introduced by a CPOE system, the present study investigated the three continuous phases of the point-of-care medication process (i.e., prescription, administration, and documentation of medications), in addition to nurses' responses to these errors. The aims of this study were twofold. First, the current study aimed to determine the incidence, causes, and types of errors associated with the prescription, administration, and documentation phases of the medication process in an ICU setting using a CPOE system. The second aim of the current work was to identify the relationships between prescription errors that occurred using a CPOE system in the different phases of the medication process and the nurses' responses to these errors.

## Methods

The present research employed a prospective observational design that involved retrospective medical chart reviews. This work was performed after acquiring permission from the IRB of Inha University Hospital (Permission #: 2008-87). With the approval, we received written informed consent from 38 nurses to participate in this study. For retrospective medical chart reviews, which were also approved by the IRB of Inha University Hospital, patient prescriptions were retrieved and then anonymized and de-identified prior to analysis.

### Study Site and Setting

The present study was conducted in two surgical ICUs (with 10 and 14 beds) of an acute care tertiary teaching hospital in Korea with 950 total beds, covering general medical, surgical, and specialty care, including oncology. The two ICUs had a total of 41 registered nurses working three shifts per day, a staff physician as manager, and three internists. Patient severity levels were measured using the Patient Severity Classification developed by the Korean Association of Critical Care Nurses in 1992. Past research has demonstrated the Patient Severity Classification scale has adequate validity and reliability based on the Acute Physiology and Chronic Health Evaluation score [8].

Medication orders in the ICU were entered by the physicians taking charge of a patient's care (mainly residents) in each medical department or internists of the ICU. The hospital had a pharmacist prescription review process for inpatients' prescriptions. Medication administration was documented in an ICU flow chart as a log of all medication-related activities. The current study was conducted between May 1, 2008 and February 30, 2009.

### Characteristics of the CPOE system

The hospital installed the first CPOE system of Medtrak (Sydney, Australia) for inpatient, outpatient, and ICU settings in 1996. It was revised and customized through partnership with a local IT solution company in 2001. Since then, the revised system was used. With the CPOE system, the user group had defined a medication administration protocol for drugs that are frequently prescribed in the ICU; this protocol was used in the training of in-house staff and nurses. The CPOE system had a function for checking drug–drug interactions based on recommendations of the Korean government for drug utilization review purposes (Health Review and Assessment Service) [9]. The CPOE system also displayed the patients' drug and food allergy information captured by nurse history-taking. During the study period, other medication-related decision support functions and electronic health records (EHRs) were not implemented. The CPOE system was checked regularly for new or modified medication orders. Nurses were made aware of any changes via notification pop-up windows on the order-retrieval screen. High-volume medications were administered at 0300, 0900, 1500, and 2100 hours (mostly at 0900 hours).

### Measurements

The primary outcome measure was the error incidence of error-prone medications in the prescription, administration, and documentation phases. The secondary outcome measure was the rate of nurses' interception through compliance with the local medication administration protocols regarding prescription errors.

The error-prone medications were defined from a literature review and the site's local experience. We adopted the “high-alert medication” list generated by the Institute for Safe Medication Practices as medications associated with an increased likelihood of errors [4]. This list was compared with the local use patterns identified by analyzing medication orders prescribed in the ICUs for the past year. The top ten most frequently used medications via intravenous (IV), intramuscular (IM), and subcutaneous (SC) routes were chosen, of which eight drug categories were high-alert medications and two were diuretics and corticosteroids. The eight high-alert drug categories were adrenergic agents, calcium, digoxin, insulin, lidocaine, heparin, magnesium, and potassium chloride. Only these targeted medications were analyzed.

### Observations and Data Collection

**Figure 1:**
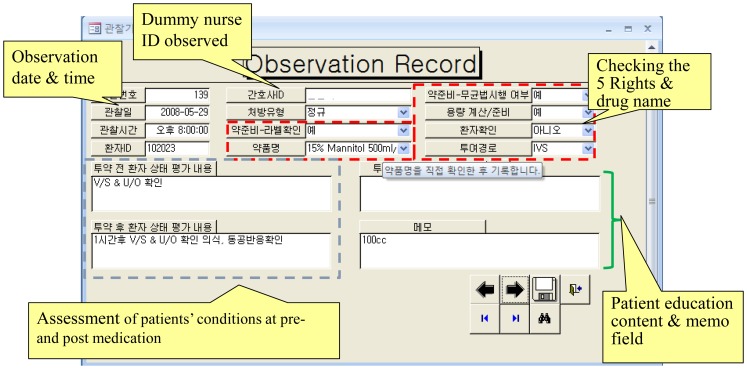
Sample screenshot of the MedObs data collector (a data input form for overt observation).

Observations were conducted on three randomly selected days per week between 0700 and 1800 hours. Six research nurses including authors (I.C., Y.J.C., and M.H.) were involved; each was assigned one of four different roles: direct observations (two nurses), order review (one nurse), chart review (one nurse), and error judgment and classification (two senior nurses). Data were collected using the MedObs data collector, a database program developed by the authors with Microsoft Access 2007. MedObs was structured into four sections that outlined what data should be captured for each role, the reference criteria for each item, and how to use the program (Fig. 1). These functions are described in detail elsewhere [10]. For retrospective medical chart reviews, patient prescriptions were anonymized and de-identified prior to analysis.

The research nurses participated in an on-site training session involving a description and demonstration of the direct observation technique, simulated medication administration scenarios, and ten real medication doses with practice data collection. A high level of agreement was reached for observational data (*κ* = 0.90). Written informed consent to participate in this study was obtained from 38 nurses after they were informed about the purpose of the study. The MedObs collector approached one nurse preselected randomly and observed her medication. Observations were conducted between May 1 and August 30, 2008, and the prescription data and medication administration records (MARs) were collected between May 1, 2008 and February 30, 2009. MedObs was used in all of the observation and chart review procedures. The data collected from observations and chart reviews were examined independently by two senior researchers using predefined error categories. Any disagreements regarding the classification were resolved by discussion.

### Definition and Classification of Medication Errors

**Figure 2:**
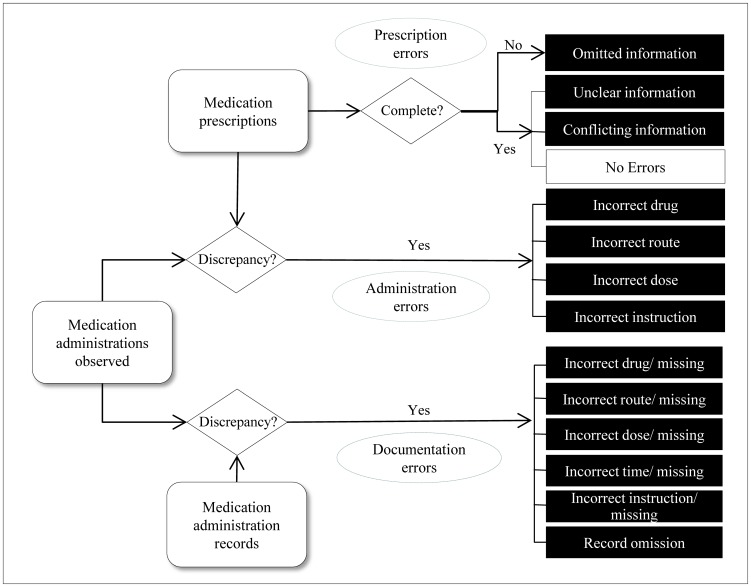
The scheme indicating the definition and classification of medication errors by clinical phase.

Prescription, administration, and documentation errors were defined by adapting previously reported definitions [11]–[13]. Fig. 2 shows the analysis scheme used in the present study for three phases. A prescription error referred to omitted information, unclear information, or conflicting information. Omitted information included incomplete prescriptions such as omission of diluent (IV mix fluid), dose, route, frequency, or instruction. Unclear information included duplications of the same order in a particular day or not specifying the total dose. Conflicting prescriptions included mismatches of a drug form and route, such as furosemide (20 mg) 3 ampules QD (*quaque die*, every day) ordered with normal saline (500 ml), but with the route given as IM. Administration errors were defined as any discrepancy between the prescriber's error-free medication order and what was actually administered to a patient. Administration errors were divided into the following categories: incorrect drug, route, dose, and instruction. Documentation errors referred to the discrepancy between what was observed and the MAR in the ICU flow chart.

### Analysis

We compared the incidence of medication errors by error category for each phase and conducted a stratified analysis of our sample for prescription and administration errors to determine relevant causes. Incidence and comparison are presented as counts with percentages and rates.

## Results

During the four-month observation period, 503 patients were admitted to the setting. Their severity levels were as follows (a higher level indicates a greater severity): levels 1 and 2, *N* = 37 (7.2%); level 3, *N* = 13 (2.7%); level 4, *N* = 147 (29.3%); level 5, *N* = 300 (59.6%); and level 6, *N* = 6 (1.2%).

For clinicians, 36 (87.8%) of the 41 full-time registered nurses working at the two ICUs participated. Their ages ranged from 23 to 39 years and mean age was 26.1 (SD = 0.4) years. The nurses had worked in the hospital and in an ICU for an average of 3.8 and 3.5 years, respectively. Among the nurses, the current working periods ranged widely, from 1 month to 16 years. Ten of the nurses had less than one year of experience in the ICU setting. The medication orders were entered by 33 in-house staff (9 internists and 24 junior residents).

In total, 614 nurses' interventions were observed. These were matched with 534 prescriptions, of which 286 (53.6%) were contained 360 errors. Frequency stratification according to error category (Table 1) indicated that 337 (93.6%) could be categorized as omitted information (most frequently route or diluent fluid omissions), followed by instruction omissions (e.g., no fluid infusion rate or designation of the dose form or type), dose omissions (e.g., missing entries for the total volume or dose volume), and frequency omissions (e.g., no documentation of the infusion interval). Unclear information and conflicting information errors were 16 (4.4%) and 7 (1.9%), respectively.

**Table 1:** Incidence of prescription errors by error category and nurses' responses on the medication administration protocol.

		Number (%)		
Prescription error category		Prescription errors	Nurses' responses on the protocol	
			Compliant	Noncompliant
Omitted information	Route†	261 (72.5)	255	6
	Diluent	56 (15.6)	55	1
	Dose†	6 (1.7)	5	1
	Frequency	5 (1.4)	5	0
	Instruction†	9 (2.5)	4	5
Subtotal		337 (93.6)	325	13
Unclear information	Dose	13 (3.6)	5	8
	Diluent	2 (0.6)	2	0
	Frequency	1 (0.3)	1	0
Subtotal		16 (4.4)	8	8
Conflicting information	Route mismatch	7 (1.9)	7	0
Total		360 (100.0)	339 (94.2)	21 (5.8)

† This category was counted concurrent with other categories.

The 286 prescription errors were reviewed to assess how nurses responded to these errors. About 94% of these errors (*N* = 339) were intercepted by the nurses (Table 1). Among the prescriptions with omitted information, 324 of the errors (96.2%) were administered complying with the medication administration protocol. However, six errors with route omission involved administration via the incorrect route. With regard to unclear information, eight prescriptions resulted in the administration of overdoses of furosemide, and crosschecking communication with physicians was not observed. However, ten administration errors, comprising two unclear diluents, one duplicated prescription, and seven route mismatches, resulted from following the medication administration protocol.

The incidence of administration errors was assessed for the 248 prescriptions, which were complete and correct with no errors. These prescriptions were observed through 306 administrations, and 58 administration errors (19.0%) were identified. Table 2 lists the errors by category and order type: routine or verbal order. Incorrect route errors were identified in 31 administrations, and 15 were dose errors in which the dose administered was at least 10% greater than that prescribed. The dose errors were similar for both order types. Incorrect drugs were given in three cases (5.2%). Rapid-acting insulin was prescribed instead of regular insulin (or vice versa); all of these were given as verbal orders.

**Table 2:** Frequency of administration errors by error category.

Administration error	Number (%)		Subtotal
category	Routine order	Verbal order	
Incorrect drug	0	3 (5.2)	3 (5.2)
Incorrect route	5 (8.6)	26 (44.8)	31 (53.4)
Incorrect dose	7 (12.1)	8 (13.8)	15 (25.9)
Incorrect instruction	9 (15.5)	0	9 (15.5)
Total	21 (36.2)	37 (63.8)	58 (100.0)

The medication documentation of 248 correctly performed administrations was reviewed. Of the eligible documentation records, 205 (82.7%) contained a total of 257 recording errors. A breakdown of the errors by error category and type (Table 3) revealed that the four most common errors were missing instructions (65.0%), missing route (12.5%), incorrect time (7.8%), and incorrect dose (5.4%). Frequently omitted instructions were the infusion rate and diluent fluid. Nine administrations (3.5%) were not recorded at all.

**Table 3:** Frequency of documentation errors by error category.

Documentation error category	Number (%)		Subtotal
	Incorrectly recorded	Not recorded	
Drug	5 (1.9)	0	5 (1.9)
Route	3 (1.2)	32 (12.5)	35 (13.7)
Time	20 (7.8)	7 (2.7)	27 (10.5)
Dose	14 (5.4)	0	14 (5.4)
Instruction	0	167 (65.0)	167 (65.0)
Omission	9 (3.5)		9 (3.5)
Total	257 (100.0)		

**Figure 3:**
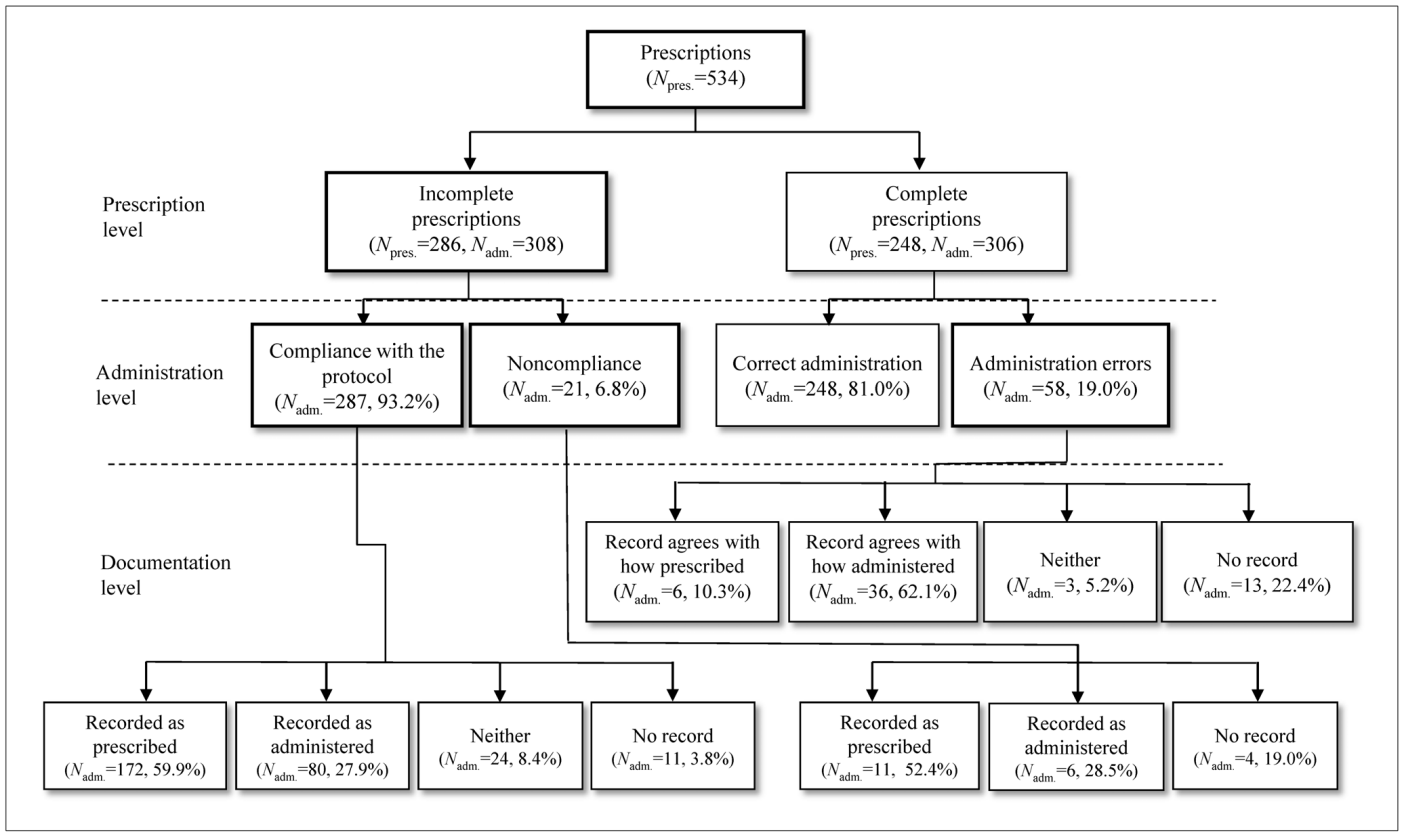
Stratification of prescription errors and administration errors.

We reviewed the documentation of 308 administrations of medication orders with prescription errors. Out of 287 correctly performed administrations, 59.9% were recorded as prescribed, 27.9% were recorded as administered, 8.4% were neither, and there was no record for 3.8% (Fig. 3). The records of the 21 administrations classified as noncompliant exhibited a similar pattern to those that were fully compliant; 52.4% were recorded as prescribed, 28.5% were recorded as administered, and for 19.0% there was no record. However, this pattern was different from what was found for 58 administration errors in correct prescriptions; 10.3% were recorded as prescribed, 62.1% were recorded as administered, 5.2% were neither, and for 22.4% there was no record.

## Discussion

Over half of the prescriptions examined in the present study included at least one error, although 94% of these were information omissions such as the route or diluent fluid. Most (93%) of the prescription errors were intercepted by nurses following medication administration protocols, but the remaining 7% reached patients. Administration errors occurred for almost one-fifth of the administrations, of which two-thirds were verbal orders. Those incorrect verbal orders entered later into the CPOE resulted in false positive administration errors. Documentation errors were also high and closely related with prescription errors. Together, these data demonstrate that error rates persist despite the use of a CPOE system. This, in turn, has potential to harm critically ill patients in an ICU.

### Prescription Errors

More than 50% of the prescriptions included at least one error, despite the use of a CPOE system, which is higher than the rate of 12% found in an outpatient setting [13]. There are two possible explanations for this high error rate in the present study. First, although the CPOE system had several medication decision support functions, such as drug–drug interactions and drug allergies, the system might be more vulnerable to information omissions and insufficient for basic functions such as consistency checking for prescription attributes, which was the main reason for the high rate of prescription errors. These errors were intercepted frequently and had a relatively low potential for harm. However, instruction omission such as IV infusion rate or duration of high-risk medication has great potential for harm. Additionally, more than half of the unclear information on dosages was not intercepted.

CPOE systems have been widely viewed as being crucial for reducing the rate of prescription errors, which is the largest identified cause of preventable adverse drug events [14]–[16]. However, mixed results have been demonstrated for CPOE systems. One study that tested the CPOE systems of 62 hospitals in the U.S. identified a very large variation (10–82%) in the detection rate of medication orders judged likely to cause serious harm to patients [17]. It is important to ascertain whether the actual CPOE systems implementations are achieving goals. Currently in the U.S., thanks to the Federal public policy efforts to incentivize health IT use, renewed attention and focus on health IT-related errors, such as malfunctions during use, incorrect use by someone, or incorrectly entered, displayed, or transmitted errors, has been highlighted. The health IT industry has been encouraged to fix and improve them [18]. End-users are also recommended to monitor and review how the systems are used internally and to measure patient-safety sensitive adverse events and medical errors regularly, related to information technology. This present study was a useful opportunity to review and improve the hospital's system.

The second possible explanation for the high error rate found in the present study is that the physicians may not have sufficient knowledge of the correct use of the system for the medication protocols. Consistent with this notion, the present study found that diluent fluid omission was the second most frequently omitted information. The most frequent omission was route attribute, which could be associated with taking shortcuts, using default selections of drug attributes, or habitual omission assuming that the route attribute is self-explicit with drugs. Furthermore, the physicians who entered the orders were mainly in-house interns or junior residents; thus, although it was assumed they were knowledgeable regarding the use of CPOE system and medication protocol, this might not have been the case. To decrease these errors, an educational approach and a better system are needed, with the goal of increased attention to issues of patient-safety sensitive functions of CPOE. Health informatics academics argue that the health care industry is relatively early in its evolution but far less than 50% of users are familiar with EHR technologies [18].

ICU systems are clearly much more complex than inpatient and outpatient systems since more drugs are used and most of those drugs are administered intravenously. Cross-referencing between prescriptions and administrations in the present study indicated that nurses played a crucial role, intercepting 93% of the prescription errors, which is higher than the rate of 86% reported by Leape et al [19]. Our results show that most of the prescriptions with omitted information such as diluent or route were corrected by nurses ensuring compliance with the hospital's ICU medication protocol. Considering that CPOE is a communication tool to deliver information between a physician and nurses administering medication, any information omission could cause misunderstanding and mistakes. A prescription should be complete and explicit for nurses as well as prescribers.

To prevent prescription errors, our findings support the need for qualified and trained nurses, more staff physicians (intensivists) working in an ICU, and a better CPOE system. A better system could integrate medication protocols into the CPOE system as a form of clinical decision support to prevent prescription errors.

### Administration Errors

The rate of administration errors found in the current study (19.0%) was higher than rates found in previous research [1], [20]. Specifically, one study in which a pharmacist was closely involved in the process of drug administration in a U. S. hospital reported a rate of 3.3% [21]. Another study found that the rate of medication errors in a medical ICU were 19.7% before and 8.7% after the implementation of bar-code-assisted medication administration [22]. This wide variation in medication error rates is due to the ratio of IV to PO (*per os*, by way of the mouth) doses and the use of diverse methods of data expression within the literature, such as the percentage of total opportunities for error or separate error rates for each phase of the medication process. The inclusion of only error-prone medications and the exclusion of PO medication in the current study might mean that the administration error rate of 19% is not actually much higher than rates found in previous research. However, given that the alert level and frequency of use of the medications were categorized as high, such errors could be quite influential.

Our examination of the order types of administration errors revealed that approximately two-thirds (37/58) of the errors occurred in association with verbal orders, with incorrect route errors being particularly prevalent. This implies that verbal orders, which constitute a relatively common part of the workflow in the ICU setting, are associated with a higher risk of administration errors. Specifically, those drugs that are in the form of liquids that can be administered either IV or SC/IM (e.g., furosemide, heparin, and methylprednisolone) were commonly associated with route errors. In the current study, verbal orders were communicated between clinicians in person or by telephone, and the prescriptions were entered into the CPOE system by the in-house staff or other delegated internists after the administration had been performed. Thus, it was unclear whether the recorded errors were due to miscommunication or inaccurate recollection by the recording physician. However, in the current study, all observers were asked to collect clinical-context data related to communications pertaining to medication between clinicians, and they reported no discrepancies between them except in one case. Furthermore, one interesting finding was that the documentation pattern differed between the corrected administrations of prescription errors and administration errors. More than 60% of the corrected administrations were recorded as prescribed rather than as administered, which were explicit documentation errors. For a prescription with omitted or incorrect information, that information was more likely to be omitted or incorrect in the administration document, even though the administration was performed correctly. It is not clear whether this pattern was relevant to the staff being concerned about leaving discrepant information between prescriptions and MARs or simply copying the prescription, but it resulted in no one knowing the correct dosage or route for a particular drug being given to a patient. In contrast, only 10% of administration errors were recorded as prescribed rather than administered, whereas 62% of administration errors were recorded as administered. In turn, this means that the nurses were more confident on medications classified as administration errors rather than those classified as prescription errors. These findings imply that the prescriptions entered later into the system were more likely to be incorrect than prescriptions before the administration phase, which were regarded as definite administration errors in the MAR.

Koppel and colleagues [23] are the only other authors to have discussed the immediate verbal order problem. The authors discussed the “now” (i.e., immediate) order as an example of medication errors caused by CPOE systems. These problems can be eliminated by clearly defining the verbal ordering process, integrating it into the CPOE system, and implementing consistency checking between the CPOE and the MAR. These functions alone could have contributed to a 64% decrease in the administration errors observed in the present study.

### Documentation Errors

Documentation errors have received less attention from researchers than prescription and administration errors. This lack of attention may be due to medication documentation errors being regarded as unimportant or not causing direct harm to patients. Few studies have explored medication documentation errors by nurses despite the introduction of a sixth right (“*right documentation*”); hence, little is known about this type of error. However, maintaining an accurate MAR is essential for both the safety and quality of care, since this record serves as a log of all medication-related activities and as a reference for the team of health providers [24]. Documentation errors can also increase the rate of false-positive administration errors when only employing a chart review method in studies of adverse drug events.

A study found that the documentation error rate increased from 13.1% to 26.7% after an electronic MAR (eMAR) intervention and most of errors were recording omissions [22]. In our sample of handwritten records, recording omissions represented only 3.5% of more than 80% of documentation errors that included a high rate of omitted information. This finding implies that although the simple adoption of a system may reduce documentation errors, the incidence of new types of documentation errors may increase. According to a study by Moreland et al., the level of satisfaction with the idea of eMARs was lowest among nurses in ICUs, although that satisfaction improved over time after they were actually implemented [25]. Those authors noted that the eMAR system did not consider the workflow for specific medication orders that occur frequently among critically ill patients, such as numerous immediate, one-time, and as-needed medication orders. An eMAR system should be able to handle verbal orders effectively so as to reduce the rate of documentation errors.

### Limitations

This study had several limitations. First, we did not obtain information about whether the medication errors caused actual harm to patients or about error severity, because we focused on the errors occurring during the medication-use process and the nurses' responses to prescription errors. Second, a single-site design was used; thus, it may not be possible to generalize the findings to other institutions that employ different processes for medication delivery, that use other forms of technology to prevent medication errors, or that have a clinical pharmacist available for consultation. The current research setting employed a pharmacist prescription review process, but it did not observe pharmacy intervention due to a shortage of clinical pharmacists. Continuing advances in technology mean that ICUs are moving away from handwritten documentation systems and toward eMAR systems. However, many ICUs continue to use paper-based flow charts and specialized medication charts for intermittent and continuous medications. Our findings could therefore benefit hospitals using CPOE systems with handwritten MARs or those considering adopting an eMAR system. The third limitation was that the night shift was not included in the observation time frame, which restricted the medication doses observed. Furthermore, the category of clinical appropriateness of drug doses in the prescription phase was not used in this study (with the exception of some clear-cut cases) because many cases were controversial due to the high severity of the patients' conditions. This might have contributed to a reduced rate of prescription errors. Finally, this study may have been susceptible to the limitations associated with direct observation (e.g., the observation effect). However, discussions during the observers' training sessions indicated that by the fifth day into the observation period, nurses were no longer affected by being observed.

## Conclusions

Despite the promise of medication error rate reduction of CPOE systems, we found high prescription and administration error rates. A significant portion of these errors were intercepted by nurses. However, the high rate of errors showed the need to monitor and measure patient safety sensitive adverse events related to CPOE use in hospitals. In addition, more attention to incomplete prescriptions with information omission, which are critical for error-prone medications and to prevent miscommunication between physicians and nurses misleading them unintentionally, is required. These efforts could guide us to a better CPOE application that integrates both verbal order processes and medication protocols.
